# A scoping review of how the candidacy framework has been used in research on access to general practice

**DOI:** 10.1177/13558196251406207

**Published:** 2025-12-16

**Authors:** Carol Sinnott, Akbar Ansari, Evleen Price, Sarah Ball, Stephanie Stockwell, Jessica Dawney, Jennifer Newbould, William D. Phillips, Jake Beech, Hugh Alderwick, Mary Dixon-Woods

**Affiliations:** 1 572461 THIS Institute, University of Cambridge, Cambridge, UK; 2 89908 RAND Europe, Cambridge, UK; 3 9058 The Health Foundation, London, UK

**Keywords:** Candidacy, general practice, scoping review, health services research, access to healthcare

## Abstract

**Objectives:**

Access to general practice is a pervasive health services concern. A tendency to conceptualise access narrowly in terms of supply of appointments may frustrate identification of better solutions. The Candidacy Framework offers an alternative conceptualisation of access as a dynamic and contingent process. We aimed to identify how the Candidacy Framework, and each of its seven features, has been applied and critiqued in research in general practice.

**Methods:**

We conducted a scoping review involving a search across four databases to identify general practice articles, editorials, books, and theses that applied the Candidacy Framework. Included studies underwent data extraction and findings were analysed descriptively.

**Results:**

Of 12,759 records screened, 73 studies published between 2007 and 2024 were included in the review. The Candidacy Framework was predominantly used in designing research or supporting interpretation of research findings. Sixty-seven papers explicitly used at least one of the seven features of candidacy; ‘navigation’ was the feature most mentioned and ‘operating conditions’ least. Candidacy appeared particularly helpful for: (1) exploring healthcare staff-patient interactions; (2) understanding barriers and enablers to accessing care; and (3) exploring complex access challenges faced by disadvantaged groups. Critiques of the framework focused on perceived linearity, lack of acknowledgement of the potential for multiple candidacies, and a need for more emphasis on contextual influences beyond local operating conditions.

**Conclusion:**

The Candidacy Framework is a useful approach for understanding access to general practice and may help in generating actionable solutions but may be enhanced by further customisation for the specifics of this setting.

## Introduction

As in other countries worldwide, access to general practice in England is a growing concern for all stakeholders, but especially for patients, who increasingly report difficulty booking appointments, longer waiting times, and interactions that do not meet their needs.^1,2^ Multiple and varied efforts have been made to address these problems, ranging from remote consultations to universal triage of appointment requests, but they have been typically modest in their effects, and may generate unintended consequences, for example on continuity and coordination of care.^3–5^

While many approaches for understanding access—a complex and somewhat contested term—have been developed in the healthcare research literature,^6–8^ one challenge in securing more effective improvement is the dominance in public discourse and debate of a narrow view of access, largely focused on the number or timeliness of appointments.^2^ One alternative to this “supply”-dominated conceptualisation is to understand access using the Candidacy Framework, which seeks to account for the multiple influences on access and how they are structured. First published in 2006, it broadly attends to “the ways in which people’s eligibility for medical attention and intervention is jointly negotiated between individuals and health services”.^9^ The framework comprises seven features: identification of candidacy, navigation, permeability of services, appearances at health services, adjudications, offers and resistance, and operating conditions (See Box 1).

Originally developed based on an analysis of the literature on how vulnerable populations in the United Kingdom access healthcare,^9^ the Candidacy Framework and its features have increasingly been used as a way of understanding access to general practice specifically. A recent review examined how the literature on access to general practice might benefit from a candidacy lens.^10^ The relative strengths of the framework include its explicit patient-centredness, its focus on access throughout the entire patient journey, and its attention to issues of structural power and culture. However, no overview or synthesis has yet been published of the growing literature on how the Candidacy Framework has been used or applied in the academic literature on general practice. In this article, we seek to address this gap.

## Methods

### Review question

The aim of this work was to review published literature that had applied the Candidacy Framework to research on general practice. Specifically, the review was designed to address the following:

(1) How has the Candidacy Framework been applied and critiqued in studies on general practice?
(2) How have studies using the Candidacy Framework in research on general practice considered each of the features of the framework?

Given the broad scope and exploratory nature of our aim, a scoping review was used because of its value for identifying how a concept has been used in the literature, and mapping and summarising a wide range of evidence from any research methodology.^11^ Our review incorporated a systematic multi-component search of the academic literature; screening of records for eligibility for inclusion in the review; extraction of relevant information from included papers; and a descriptive synthesis of the findings relevant to each of the research questions.^11,12^ Presentation of results adheres to the PRISMA-ScR reporting guidelines (Online Supplemental, material 1).^12^

### Search strategy

As the term Candidacy can have varied meanings, we used a systematic multi-component search to identify articles that cited and/or applied the concept of Candidacy as defined by Dixon-Woods et al.,^9^ including but not limited to its application in general practice.

This involved:

- Searches in Web of Science (WoS) and Scopus for citations to Dixon-Woods et al.^9^ or one of three seminal papers on candidacy by Mackenzie et al.^13–15^ up until 26 September 2022.
- Using the ‘related documents’ and the ‘related records’ search functions in Scopus and WoS to identify articles that share references with Mackenzie et al.^13–15^ and include the term “candida*” in any field.
- A wider literature search in WoS and Scopus for papers that use the term ‘candidacy’ in the same sense as Dixon-Woods et al.^9^ (combining “candidac*” with terms relating to healthcare/access to services).
- A Google Scholar search for articles citing Dixon-Woods et al.^9^ that included the terms ‘candidacy’ or ‘candidacies’.
- An additional search for potentially relevant theses and dissertations published since January 2006, using the Open Access Thesis and Dissertations platform, on 4 October 2022.
- The original searches were run between 21 and 26 September 2022. All searches were rerun on 12 November 2024 to include the period between September 2022 and October 2024.

After deduplication of records, references were managed using Endnote. A full description of the search is presented in the Online Supplemental, material 2.

### Screening for eligibility

Screening of title and abstract was undertaken in two rounds, first to identify those focused on ‘Candidacy’ and then to narrow down to those focused on general practice. The inclusion and exclusion criteria are shown in Table 1. In the first round, screening was undertaken by three reviewers (SB, SS and WP). In the second round of screening, two reviewers (SS and JD) initially screened the same sample of 54 records and compared results to ensure consistency. The remaining records were split between the two reviewers. Disagreements were resolved through discussion with other members of the review team. Reviewers erred on the side of inclusion. A single reviewer (SB) screened full texts of potentially relevant articles. Articles with only brief mentions of Candidacy were excluded. Some articles were excluded during the extraction process when their limited content relating to Candidacy became apparent. The same process was followed for the updated search in October 2024 (reviewers CS, AA and EP).

**Table 1.** Eligibility criteria.

Inclusion criteria	Exclusion criteria
Academic articles, editorials/opinion pieces, books/chapters and theses referencing candidacy and which describe use of the candidacy concept or framework, including all or any of its seven stages, in research on access to general practice^a^	Articles that
Papers	• Use candidacy in a different sense from that defined by dixon-Woods et al. 2006. For example
• Reporting on adults and/or children	- Lay epidemiology
• Published subsequent to Dixon Woods et al. 2006	- Surgical/treatment candidacy
• Written in English	- ‘Candidate’ theories, concepts, mechanisms or causes
	- Political candidacy
	• Cite Dxon-Woods et al. 2006 for the methodological approach of critical interpretive synthesis rather than candidacy concept
	• Report studies that were conducted
	- In a low- or middle-income country
	- Outside of the general practice setting
	• For which the full text was not readily accessible
	• Took the form of document types other than research articles, theses and books e.g
	- Grey literature reports
	- Protocols
	- Practice guides
	- Conference proceedings
	- Journalistic/news articles

^a^For the purposes of article screening, general practice was defined as: a community-based point of first-access care for undifferentiated health conditions offered across the lifespan. The focus was on access to care provided in the general practice setting, rather than to a GP specifically and includes access to other health professionals or services provided in this setting.

### Extraction

Two reviewers (SS and JD; CS and EP for update) extracted information using a customised data extraction template, which captured information on: the topic of the paper (study population, geographic focus); how the Candidacy Framework was used; which features of the framework were mentioned specifically and high-level findings relating to these features; and critiques of the framework, including authors’ reflections on its strengths and weaknesses when applied to general practice and their suggested modifications to the framework. Reviewers recorded study findings in line with the authors’ conceptualisation (rather than their own interpretation). As is usual in scoping reviews,^11^ we did not formally assess or exclude papers based on quality, but reviewers did record their reflections on the quality of included papers. To ensure consistency, both reviewers initially extracted information from three articles and compared their results, with remaining articles split between them.

### Synthesis

Characteristics of included articles were summarised in a table. The extracted findings were synthesised and summarised descriptively to provide an overview of the use and critique of the concept of candidacy in research on access to general practice and to identify key characteristics of each of the seven features of the framework within this context.^11^ An analysis workshop involving members of the review team was conducted to aid the synthesis of findings.

## Findings

**Figure 1.**
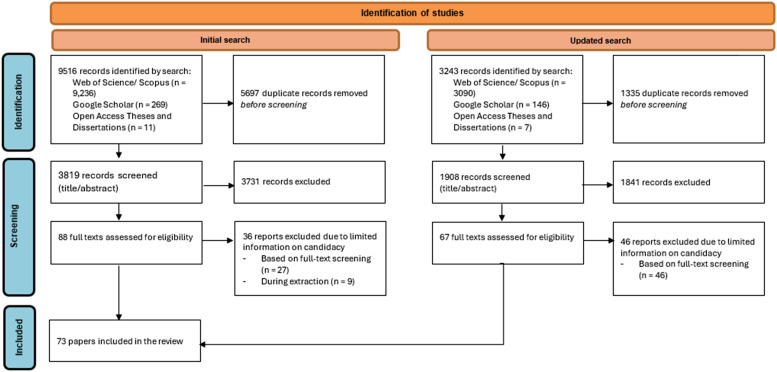
PRISMA flow diagram.

Of 12,759 potentially relevant citations identified, 73 papers were included in the review (Figure 1). All were published between 2007 and 2024. The majority (*n* = 56 papers) described empirical research, 11 were reviews, and the remaining six were theses. The papers included in our review used data from the UK, USA, Canada, Norway, Australia, Denmark, Malta, Israel, New Zealand and the Netherlands. Approximately half (*n* = 36) focused on marginalised, stigmatised or vulnerable populations. A further 21 papers focused on people with specific health conditions (e.g. chronic respiratory disease). Five focused on conditions in specific age groups, while seven involved people using specific services, including those offered but not attending care. The remaining papers included a policy analysis, an exploration of gender differences, a study of the impact of digital technologies, and a review of previous literature on access. Full study characteristics are presented in Online Supplemental, material 3.

Although we did not formally assess quality, methods generally appeared to be clearly described in the included papers. 11 papers^16–26^ did not self-report any limitations, but the remainder reported a range of limitations of their studies, including those associated with response rates and small samples,^27–62^ issues of representation and limited generalisability,^5,42–44,49,59,62–74^ recall bias,^39,45,54,59,63,74^ problems with data linkage or data not capturing changes over time,^36,40,48,63,75,76^ and researchers’ status as clinicians.^27,77–79^ Authors of some reviews reported that lack of contextual information and description of interventions made it difficult for them to compare or synthesise studies.^54,68,69,80–83^

We present the findings of our review of these studies below in two parts, first considering how the Candidacy Framework has been used and critiqued generally in studies on general practice and then offering an analysis of how the studies included in our review have considered each of the features of the framework.

### Use and critiques of the candidacy framework applied to general practice

In the studies we included, the Candidacy Framework was used in a number of ways, for example to support the organisation of data,^19,49,83^ as an analytical or interpretive tool,^10,26,50,60,65,66,69,71,72,74,75,79,80,84,85^ to support development of conceptual frameworks,^5,21,47,64,67,68,76^ or programme theories,^70^ to support development of hypotheses,^73^ or to guide analysis of survey findings.^59^

Candidacy was frequently deployed alongside other theories and concepts^5,21,24,25,38,45,48,52,53,57,65,67–70,72–74,76,79,84,86^ including those that were specific such as fundamental causation,^69^ structural vulnerability,^57^ or socio-technical theory^5^ as well as more general concepts such as trust,^70^ person-centredness,^52,70,73^ and burden of treatment.^72^

Thematically, the framework was used to explore patients’ engagement with services^24,40,41,51,66–69^; influences on, barriers to, and enablers of access to healthcare, such as previous experiences of care^27,44,48,65^; the work required of patients to secure appointments,^5,24,39,42,81^ and the complexities of access for vulnerable, underserved or seldom-heard groups.^19,20,39,42,43,46,49,55,57,66,67,76,81^ The framework was also deployed to explore healthcare professional-patient interactions^31,51,52,62,63,79,83^ and misalignment between patient-side and health service-side views on access.^26,34,44,84^ Some studies used it specifically to understand access to preventative care,^57^ to explore general practitioners (GPs’) gatekeeping role in relation to other healthcare services,^47,62,74,81,82^ to examine the impact of health policy^22^ and as a unifying concept to describe access and continuity.^75^ More recently, the framework has been used to explore the use, and potential exclusionary tendencies, of digital technology in general practice, leading to the development of the concept of ‘digital candidacy’.^5^

Among the included papers, 32 offered some form of critique of the Candidacy Framework.^5,10,22–26,40–43,45–48,52–54,57,59,62–64,66,72,76,81–86^ The framework was valued for not being ‘too abstract’, helping to generate explanations that were testable,^73,81^ for its utility in guiding quantitative research,^59^ for helping to explain why some policy interventions might not work as well as expected,^22^ and for aiding the recognition and interpretation of important aspects of access that might otherwise go unnoticed.^63^ In capturing how access and eligibility for services are influenced by socio-cultural, economic, and organisational factors, the framework was seen to be helpful in revealing the complexity of accessing healthcare for specific populations of interest,^39,41,42,81^ for example by including samples beyond that of vulnerable groups for which it was originally designed,^10,47^ such as those with complex chronic health conditions.^45,47,64,80^

The framework was deemed useful for exploring the role of patient-professional interactions^24,31,40,51,52,62,63,83^ and explaining how previous experiences and outcomes,^45^ misaligned perspectives between patients and professionals,^48^ fear of stigma or judgement,^27,62^ and lack of cultural competence^43,44,67,85^ might all influence access beyond simple “supply” of appointments. Further, the framework was helpful in showing how patients might be knowledgeable and discriminating in their healthcare choices, and use their experiential knowledge when choosing between services,^45,67,84^ thus countering “deficit” models that locate difficulties in access in patients’ lack of information and education.

Criticisms of the framework included a perception that it is overly linear^26,40,48,53,57,85,86^ and insufficiently accounts for the many interactions that might take place between the healthcare system and patients.^24,43,48,57,64,72^ To better cater for this recursivity, authors suggested presenting the framework more cyclically^57,64,81,82^ or adding new features to link past care experiences and outcomes to future expectations and behaviours.^45,48,64^ Some studies suggested that more attention was required for illness context, individual and collective sense-making and actions that could lead to candidacy being validated, diverted, rejected or disrupted.^26,54,65^

The role of intersectionality was also seen as requiring more emphasis in the framework.^64^ For example, intersecting individual identities (e.g., the combination of socio-economic status, gender and mental health status) might diminish or enhance assertions of candidacy and the challenges patients must overcome to gain entry to the healthcare system, especially in vulnerable individuals.^15,43,57,61,64^ Multiple co-existing candidacies for different services, including those associated with multi-morbidities^40,53,57,82,85^ and changes in patients’ illness identities over time^48,64,86^ were seen as needing further space within the framework, as were tensions between patients’ cultural perspectives, social norms and practices that might not align with professionals’ Western cultural assumptions.^25,43,67^

### Findings relating to the seven features of the candidacy framework

**Figure 2.**
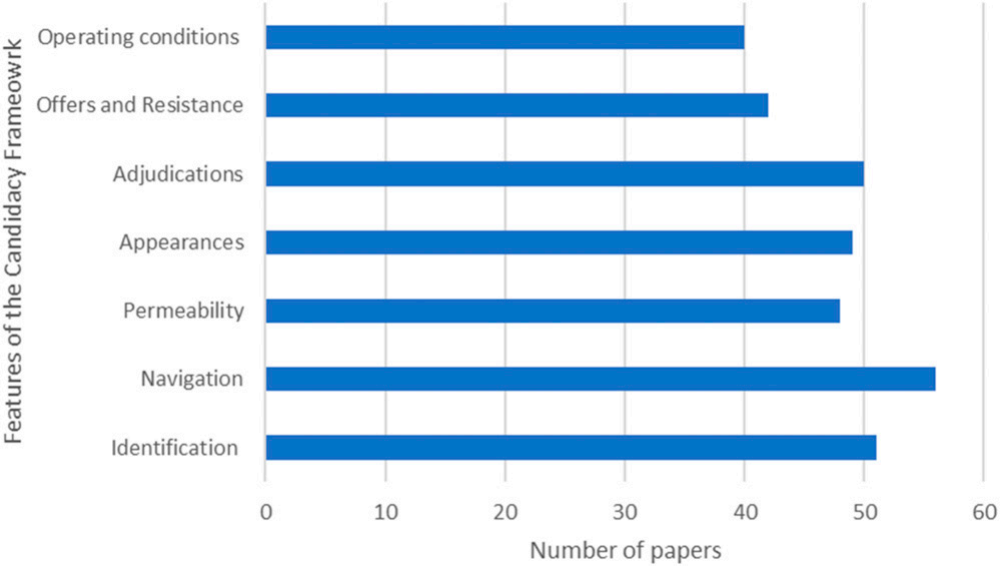
Number of papers explicitly referring to different features of the Candidacy Framework.

Of the 73 included papers, 67 specifically discussed at least one of the features of candidacy. Figure 2 highlights the number of papers that explicitly referred to each feature, showing that ‘navigation’ is the most discussed and ‘operating conditions’ the least. Some studies queried the definitions and distinctions between the features,^40,41,59,74,81^ or proposed grouping them into categories, though not always in the same ways.^43,59^ What follows is a summary of the findings relating to each of seven features individually.

#### Identification

Identification is defined within the Candidacy Framework as: “how people recognise their symptoms as needing medical attention or intervention”.^9^ Papers included in the review described the process of identification as involving patients’ considerations of the significance and impact of their symptoms on their functional status and quality of life,^18,33,34,52,62,86^ their previous history or experiences of a health problem,^47,48^ and whether their symptoms represented a “crisis”.^39,48,72^ Continuity of care, especially relational continuity, was found to support people in identifying their candidacy^53^ as were prompts or reminders from practices to attend for particular services.^55^ In addition, patients’ familiarity with the local healthcare system and the availability of self-care options and wider healthcare services were consequential.^47,48,58,63,67,76^ So too were patients’ social networks: multiple people may be involved in identification,^20,42,44,67,70,72,74,75,82,83^ while caregivers, in turn, used evidence from their own networks to make their ‘case’ for an identified need.^74^ Although family and wider community might be a source of social support, they might equally be a source of stress, guilt or shame.^43,62^ Studies characterised the role of social media and online peer groups in supporting patients to identify themselves as candidates for healthcare, for example for people with long covid,^26,54,65^ and to develop “collective” candidacy for groups of patients.^14,26^ However, some healthcare professionals believed that such resources could confuse rather than empower patients.^65^

The evidence we reviewed identified that people can have multiple personal identities (associated, for example, with sociodemographic characteristics, roles and illnesses), and that identification of candidacy was in some cases affected by the nature of the health condition itself (e.g. depression).^60,62^ Some studies suggested that identification might draw on a mixture of models about health and illness: Western biomedical explanations might coexist alongside more traditional, religious and spiritual beliefs, leading to different responses to symptoms across cultural groups and in different generations of the same groups.^43^ Language skills,^19^ lack of knowledge and poor availability of health-related information^20,42,52,59^ negatively impacted identification and multiple disadvantages sometimes intersected to compound these effects.^60^

In deciding to seek care, studies reported that patients considered not just one appointment but their utilisation of the healthcare service as a whole,^69^ as well as whether treatment was likely to be available and how (or whether) it might help.^26,69^ Their decisions were further influenced by personal views on whether they should endure so that services were left to those with the greatest need or by a “rights and access” mindset that tended to emphasise eligibility and the need to fight for it if necessary.^70,71^ Feelings of unworthiness and fear of inconveniencing others were common among various groups. For example, healthcare workers sometimes felt unable to take time off when sick,^26^ while others, for example people who inject drugs, sometimes believed they did not deserve treatment or saw their symptoms as an inevitable consequence of their actions.^18,19,41,43,57,69,76^ Self-identification of candidacy was also framed by differences in men’s and women’s caring and paid work responsibilities and access to resources such as finance, transport and personal time.^23,60,71^

Some authors suggested that the Candidacy Framework might be less useful when symptoms or conditions were poorly defined,^54,64^ with the fragility of Candidacy for symptoms that might be capable of multiple interpretations hindering identification and help-seeking.^26,46,53,64,65^ Identification was also suppressed for symptoms affected by stigma or socio-cultural influences, for example those relating to mental health, domestic violence, gynaecology or sexual health.^18,40,52,59,66–69^ Others suggested the need for a ‘pre-identification’ feature in the framework, since decisions about service use versus self-care may be made before Candidacy is identified.^53^

When general practice reaches out to patients to alert them to their candidacy for certain interventions (e.g. vaccination, health checks), it takes on some of the work of identification for patients. Reactions to such invitations were variable, ranging from gratitude or enthusiasm to scepticism and uncertainty,^70,72^ with lower engagement with preventative care or long-term illness care evident in more deprived social groups.^22,57,70^

#### Navigation

Navigation refers to how “people must be aware of the services on offer” and how using “health services requires the mobilisation of a range of practical resources”.^9^ The studies in our review typically depicted navigation as requiring competencies, resources and work that were socio-economically and culturally patterned.^44^ A key competency is awareness of the services available, while the capacity to reach them may require substantial resources and effort.^19,35,57,64,65,76^ Insufficient transport, internet and phone connectivity, childcare, language skills and finances to cover the costs of attending were all reported as challenges.^18,19,25,39,46,48,60,66,67,69,76^ The introduction of digital access platforms, apps and online triage was identified as making the work of navigation more difficult for some patients,^5,60^ as did the lack of appropriate services for a particular condition.^48,65,84^

Navigation was more difficult in the absence of culturally competent services,^18,43,67^ but benefitted from clear signposting, for example from pharmacists^51^ and from the practical support of patient advocates, practice administrative staff, social workers or third sector agencies, especially for those who needed help registering with a practice.^24,38,40,42,55,69,72,85^

Conditions that did not sit neatly within either health or social care, such as dementia, caused navigation problems as patients ricocheted between services when seeking support.^84^ While some patients, for example people experiencing addiction or mental illness, did not see general practice as the right place for them to seek healthcare^66,77^ others simply used general practice to support their navigation to the services they felt they did need.^21,47^ General practices sometimes offered enhanced or locally-agreed services, for example sexual health clinics, but patients did not always know what specific services their practice offered, especially if they moved from one practice to another.^52^ Patients’ choices about what services to use were also heavily influenced by previous experiences of care, rather than, for example, public information campaigns.^45^

#### Permeability

Permeability is defined as “the ease with which people use services”.^9^ It is strongly influenced by the alignment between the services available and users’ needs, capabilities and personal characteristics,^20,27,64,69^ may vary depending on stage and type of illness,^84^ and may be strongly linked to navigability (above).^76,83^

Although general practice is intended to be highly permeable, the papers in our review identified that a shortage of appointments created difficulties for patient access^51^ as did difficult processes for making appointments.^69^ For instance, patients often preferred making appointments by telephone, but even telephone requests could be problematic if patients had to wait on hold for long durations or had to call multiple times, with these difficulties more pronounced in those experiencing financial hardship.^60^ Problems securing an appointment at general practice made emergency care, which requires no appointment, appear more permeable to patients,^22,34,39,44,45,48,53,55,58,76,84,86^ and over time, patients who experienced difficulties getting the care they need from general practice were more likely to default to emergency services.^45,60^

Studies found that permeability was better for patients with an existing diagnosis,^44,86^ who shared cultural characteristics with healthcare staff,^19,27,44,67^ or who had others advocating for them.^55,75^ Conversely, limited access to interpreters decreased permeability for patients for whom English was not a first language, while lack of a mobile phone with credit was also consequential.^18,40,44^ Patients who were less familiar or had less trust in services^18,24,57,67,72^ and those with sensitive or complex health conditions^23,26,65,77^ appeared to experience restricted permeability, leading some to question their candidacy and return to the identification stage in a negative feedback loop.^26^

#### Appearances

According to the Candidacy Framework, “appearing at health services involves people asserting a claim to candidacy for medical attention or intervention”. [5] In order to assert a claim for a service, papers in our review found that individuals must be able to articulate the help they seek,^51,80^ and that the communication skills of both patients and clinicians influence this process.^18,20,64^

Familiarity and continuity were important features of appearances: patients did not like having to repeatedly re-establish their candidacy with different providers.^60,65,66,75^ Although in-person encounters seemed to support patient’s sense of candidacy more than telephone interactions,^70^ the warmth of in-person interactions appeared to be increasingly replaced with remote care or words on a screen that dehumanised encounters.^5^ Further, patients sometimes lost their chance to ‘appear’ through missing call-backs from practices or inopportune appointment times that clashed with other responsibilities such as work or childcare.^60^

Papers also mentioned fear of wasting doctors’ time, imbalanced power positions, being unable to advocate for one’s own needs, sensitivity to cultural and language differences and fear of repercussions of full disclosures as constraining appearances in general practice.^18,19,24,43,51,57,66^ Appearances seemed more difficult for patients when the diagnosis was unclear or when patients had a “gut feeling” but no concrete evidence of anything being wrong.^74^ Conversely, patients with known conditions had more satisfactory experiences of appearances.^26,44,65,74^ In the case of mental health conditions, some patients had symptoms severe enough to impact on their ability to present their problems and collateral histories were required.^27,35,53,55,59,66,70,72,75,76^

Gender differences were also described. Older women were reported to be less likely to discuss symptoms of depression than older men^19^ while men with rheumatoid arthritis could be overly stoic in their description of pain or discomfort.^64^ Potentially shaming issues might be introduced in subtle ways, such as framing requests for sexually transmitted infection (STI) screens as a desire for a general health check.^67^

Unsatisfactory communication with healthcare providers could lead to reluctance to re-engage or seek further support.^27,35,53,55,59,66,75,76^ Particularly in minority ethnic groups, more deferential styles of communication could restrict patients’ ability to articulate their claims for candidacy and could lead to sensitive issues being avoided.^43^ In contrast, feeling listened to without judgement was reported to be validating, particularly for those with unclear or multiple diagnoses,^70^ as was feeling ‘believed’ by healthcare professionals and listened to when suggesting potential treatment options.^54,64,67^ Some authors called for greater acknowledgement of the relational nature and power dynamics of patient-professional interactions in general practice within the framework.^24,42,46,66,69,72^

#### Adjudications

Adjudications are defined as “the judgements and decisions made by professionals which allow or inhibit continued progression of candidacy”.^9^ Most studies in our review used adjudications to depict interactions between patients and clinicians, but some applied the concept to administrative decisions (e.g. practice registration)^38,39,42^ and others used adjudications to represent any opinion or advice given to a patient, even if from friends or family.^74^

More generally, studies in our review suggested that adjudications in general practice may comprise considerations relating to the technical feasibility of providing an intervention or services, which is influenced by local resources and the conditions in which practitioners work^22,65,72,76^ and their considerations of the likely benefits of an intervention (or non-intervention).^23,26,35,36,48,53,63,66,79^ Adjudications involving GPs’ gatekeeping role in providing access to other services was also a prominent theme^59,64,65,74,82^ and there was evidence of patients and professionals ‘tinkering’ with the services available to make them fit individual patients in ways unintended by the system.^79^

Many papers described patients’ expectations not being met: for instance, desired referrals not being made or investigations not being ordered,^20,54,63^ refused sick/fit notes,^37^ insufficient discussion of preferred treatment modalities,^47,52^ and consultations ending in uncertainty about the diagnosis or problem being treated.^20,26,44,54,77^ Patients’ experience of adjudications were sometimes tainted by misaligned communication styles,^43^ misconceptions,^39^ insufficient receptiveness,^20,54^ feeling judged^26,60,66,74^ or limited training on cultural competency.^18,19,24,59,66^ These professional judgments were powerful in their impact on patients’ assessment of their entitlement to care and subsequent help-seeking^23,26,40,42,69^ with some patients feeling devalued,^27^ and their candidacy undermined.^26,62,74^ However, other patients responded to negative adjudications by building evidence of their candidacy and returning to secure their desired adjudication.^72,74^

In contrast, shared understanding of purpose, clarity of expectations and power sharing were found to facilitate trusted relationships between professional and patient, and validated candidacy.^54,64,67,70^ Continuity of care was seen to support more patient-centred adjudications in several studies.^52,53,65,66,75^ Some authors sought more explicit acknowledgement of the need for patient-centredness and concordance between care providers and patients about what the problem is and what the best solutions might be,^5,48,64^ with a feature titled ‘recognising and accepting candidacy’^52^ proposed to emphasise the local influences on adjudication and the back and forth that occurs between patients and professionals in consultations.

#### Offers and resistance

Offers and resistance refers to “the idea that people may refuse an offer of a service, medication, or other support”.^9^ Not using a service does not necessarily mean that services had not been offered,^76^ and an individual’s personal and socio-cultural situation could affect the extent to which offers were accepted or acted on.^18,19^ However, studies in our review revealed that offers were more likely to be accepted where there is trust,^18,20,63^ a respectful patient–provider relationship,^20,35,46,65^ or confidence in the GP’s scope or expertise in the matter at hand.^44,77^ How a service is framed to service users also impacts uptake of offers^81,82^ but a lack of shared vocabulary meant that certain groups struggled to understand what was on offer,^22^ potentially explaining why people with lower health literacy reported poorer experiences of care.^73^ Cultural norms could impact on patients’ ability to make changes at a personal level. For example, social norms around food in some South Asian communities conflict with dietary advice in diabetes,^43^ while the literacy and transport demands of some offers simply outstripped patients’ capabilities and practical resources.^60^

Unfavourable adjudications influenced whether offers were accepted or resisted,^42^ as did previous negative encounters with health professionals,^27,53^ perceptions of the likely effectiveness of an offer,^47^ and perceived misalignments in the focus of care.^48,70^ Some offers were accepted but modified by patients - for example, deliberately reducing their use of medications from what was prescribed to minimise their risk of potentially serious adverse effects.^64^

#### Operating conditions

Operating conditions are defined in the Candidacy Framework as “the local influences and locally specific interactions which impact dynamics between practitioners and patients”.^9^ This feature was used variably in studies included in the review. Some linked operating conditions with trust, continuity, power imbalances and other aspects of interactions between healthcare professional and patient.^27,35,48,52,53,63^ The structures and responsibilities of general practice within the health service were also framed as operating conditions, for example GPs’ role as gatekeepers for other services or settings in which financial transactions take place between patients and general practices.^51^ Others saw the availability of support from families and communities as operating conditions.^18,47^

Generally, however, operating conditions were seen to relate to wider system issues such as the availability of, wait time for, and coordination with specialist and community services.^37,40,46,72^ Deficiencies in services outside of general practice could negatively impact the relationship between patients and GPs,^37^ and even when deficiencies impacted access for patients across the board, could be perceived by some groups as an indication of hostile attitudes towards them^42,65^ and led some patients to seek services privately.^74^ Yet other papers depicted broader cultural and socio-political issues such as austerity as operating conditions.^26,65,67,70^ For example, some national policies (e.g. on immigration) were identified as disproportionately disadvantaging certain populations.^19^

While the local dimensions of “operating conditions” were agreed to be important for general practice,^44^ many authors felt that the Candidacy Framework failed to sufficiently capture the wider-ranging meso- and macro-level influences on access to general practice.^23,40–42,46,57,62,72,76,81–83^

## Discussion

This review of 73 articles published since 2007 offers an overview of how the Candidacy Framework has been used and critiqued in the academic literature on general practice. Overall, the concept of Candidacy and its features appear to have been of considerable value, helping to re-imagine access as subject to multiple, diverse and contingent influences that are socio-demographically patterned, and not a simple matter of “supply” of appointments. Our analysis of the available literature makes clear that the Candidacy Framework, even as currently formulated, offers highly practical insights into why improving access to general practice is such a difficult challenge. It explains, for example, why simply increasing the number of appointments in general practice may not “solve” the problem of access, since many other features of Candidacy shape and structure people’s experiences of access in ways that may systematically disadvantage some groups. Similarly, using the framework explains how strategies based on patients’ “deficits” of understanding – such as education – are unlikely, on their own, to change care-seeking behaviour, demonstrating instead the importance of experiences in recursively shaping candidacy.^45^ The increasing use of the Candidacy Framework in the analysis of quantitative data illustrates a growing appreciation of the benefits of explaining access in a more sophisticated and structured way.^59,73^

Some aspects of Candidacy have received much more attention in the literature than others, with “operating conditions” least likely to be given consideration. Further, while some authors interpreted operating conditions as including the organisational pressures, policy imperatives, institutional and infrastructural factors that wrap around the other features,^23,81^ others felt that greater attention on the macro-structural and social-contextual factors that affect candidacy was needed.^40,42,46,57,62,72,76,82^ This finding is relevant given that the broader operating conditions for GPs - for instance, policies on how funding is allocated, the role of GPs in the health system, coordination between services, and so on - can have a substantial impact on how general practice works and what care is available to access.^4^

The literature in our review also offered critiques of Candidacy when used in the context of general practice, suggesting several ways in which it might be enhanced or modified. Proposals for amending the Candidacy Framework for general practice include increased emphasis on: (1) the cyclical and recursive character of help-seeking behaviour, including the impacts of past experiences and care outcomes on future expectations and behaviours^24,26,40,43,48,53,57,64,72,81,82,85,86^; (2) the potential for multiple and potentially conflicting candidacies and the importance of both illness and socio-cultural identities^40,53,57,62,82,85^; and (3) the characteristics of interactions between patients and professionals that are particularly important in general practice, such as trust and familiarity, continuity, and patient-centredness.^5,24,42,46,48,64,66,72^ Additional proposed amendments to the framework depict micro-level negotiations between service-users and professionals as being embedded within their local/organisational (meso) and national/political (macro) contexts.^14,40,57,82^

A number of studies also suggested specific changes to the framework, such as changing the order or interactions between different features,^43,59^ adding new features, new components to existing features, or specific contextual factors.^26,48,52–54,64,86^ It was also evident from included papers, however, that features were being applied in different ways in different studies, with some commentaries suggesting that the boundaries between features of framework are becoming increasingly blurred as the complexity and fragmentation of general practice increases.^40,41,59,76,81,83^ These observations and proposals warrant further consideration and empirical theoretical testing, especially as general practices increasingly rely on digital technology and remote consultations to deliver care, impacting access experiences.^5^

### Limitations

While the search methodology for our review was systematic, some relevant literature may have been missed owing to the databases searched, or may have been excluded due to eligibility restrictions on language and low- or middle-income countries. The nature and focus of the included articles limited the degree to which conclusions can be drawn about the fit of the Candidacy Framework to aspects of general practice access that have not been studied. The study populations were generally narrowly defined in the articles we reviewed, with many focusing on specific marginalised or vulnerable groups with known access challenges. This, and the fact that healthcare funding, provision and structure varies in the countries represented by studies in the review, may limit the applicability and generalisability of the findings to other populations, for example in terms of specific barriers in navigation and the significance of negative adjudications. Skill-mix in general practice has increased significantly over recent years, but research on the impact of these changes is only emerging. This means that it was not possible to establish, based on the included papers, how the range of roles in general practice influences access as conceptualised in the seven stages of the Candidacy Framework and may be a valuable area for future research. We did not use a generic analytical tool such as PAGER^87^ in our analysis, instead we tailored the analysis to the specific objectives of the review. Nevertheless, our findings illustrated patterns in how the Candidacy Framework has been used, the aspects of general practice to which it has been applied, and the theoretical advances and proposed refinements made as well as generating evidence for practice and research recommendations.

### Implications for research, policy and practice

Critiques of the Candidacy Framework—which has already been frequently deployed alongside other theories and concepts—point to the potential value of applying it alongside other explanatory models used to study general practice, but questions remain on what the most suitable additional models might be. The concept of access itself may benefit from further refinement, particularly in light of recent work that has sought to problematise and transcend oversimplified definitions of access: for instance, Levesque et al.’s^7^ articulation of five dimensions of access and five corresponding abilities of populations, and Voorhees et al.’s^8^ definition of access as the ‘human fit’ between the needs and abilities of the population and the capacity and abilities of the healthcare workforce.

The significant number of papers that have used the Candidacy Framework to study access to general practice highlights the increased role that questions of access have played in the wider policy debate in recent years. The prominence of access is likely to be motivated by a variety of reasons (for instance, decreasing satisfaction with access to general practice, increasing recognition of the factors associated with access, the impact of the COVID-19 pandemic); but, against the background of general practice delivering a reported record number of appointments,^88^ it is clear that access is not a simple equation involving the supply of appointments or number of doctors per head. Getting an appointment quickly and easily certainly is an important aspect of access, but so too is the degree to which patients feel that their needs are being met and are confident in the quality of care.^89^ Accordingly, this review offers key insights into the extent to which factors outside of simply “booking an appointment” can impact and affect people’s experiences of access.

Given the wider policy concern about access to general practice, the findings here suggest that the Candidacy Framework may itself be a useful and actionable tool to support those working in general practice policy and practice to think innovatively and comprehensively about where improvements to access are needed across the entire patient journey. Indeed, insofar as any efforts to improve access may involve trade-offs between different components of access, the Candidacy Framework may be of particular use in the identification of where, how, and for whom such trade-offs are likely to occur.^1,3^

### Conclusions

Our review of a large literature which has applied the Candidacy Framework demonstrates the value of the framework in explaining the influences on access to general practice, though it may also benefit from further customisation for this context. Given the concern about access to general practice at multiple levels, tailoring use of the framework may be helpful in finding actionable, equity-focused solutions.

**Box 1. Summary of the seven features of the candidacy framework for understanding access^9^**

**Table:** 

Identification	How people recognise their symptoms as needing medical attention or intervention is important to how they assert a claim to candidacy
Navigation	Using services requires knowledge of the available services and depends on having the practical resources to use them
Permeability	The ease with which people can use services depends on how many and what kinds of criteria people must meet to use them, and on cultural and other alignments between services and individuals
Appearances	Appearing at services involves people making a claim to candidacy. It requires a set of competencies and socio-cultural alignments
Adjudications	Professional judgements about patients’ candidacy strongly influence individuals’ access to attention and interventions
Offers and resistance	Offers of care may be made that may be accepted (e.g. utilisation) or refused (e.g. non-utilisation) by individuals
Operating conditions	The perceived or actual availability and suitability of resources has a major impact on the local production of candidacy, as do other relevant operating conditions

## Supplemental Material

Supplemental Material - A scoping review of how the candidacy framework has been used in research on access to general practice Supplemental Material for A scoping review of how the candidacy framework has been used in research on access to general practice by Carol Sinnott, Akbar Ansari, Evleen Price, Sarah Ball, Stephanie Stockwell, Jessica Dawney, Jennifer Newbould, William D. Phillips, Jake Beech, Hugh Alderwick and Mary Dixon-Woods in Journal of Health Services Research & Policy

## Data Availability

As a scoping review, all synthesized data is public facing. Information on search strategies are provided in the supplementary material.*
