# Supplementary material for: A scoping review of how the candidacy framework has been used in research on access to general practice
Source: J Health Serv Res Policy. 2025 Dec 16;31(3):196–209. doi: 10.1177/13558196251406207 (PMC13263460; doi:10.1177/13558196251406207)
Supplement: Supplemental Material - A scoping review of how the candidacy framework has been used in research on access to general practice [file sj-pdf-1-hsr-10.1177_13558196251406207.pdf]

Online Supplement

1 Supplementary material 1. PRISMA-ScR reporting guideline [1]

SECTION	ITEM	PRISMA-ScR CHECKLIST ITEM	REPORTED ON PAGE #
**TITLE**			
**Title**	1	Identify the report as a scoping review.	1
**ABSTRACT**			
**Structured summary**	2	Provide a structured summary that includes (as applicable): background, objectives, eligibility criteria, sources of evidence, charting methods, results, and conclusions that relate to the review questions and objectives.	2
**INTRODUCTION**			
**Rationale**	3	Describe the rationale for the review in the context of what is already known. Explain why the review questions/objectives lend themselves to a scoping review approach.	3
**Objectives**	4	Provide an explicit statement of the questions and objectives being addressed with reference to their key elements (e.g., population or participants, concepts, and context) or other relevant key elements used to conceptualize the review questions and/or objectives.	3
**METHODS**			
**Protocol and registration**	5	Indicate whether a review protocol exists; state if and where it can be accessed (e.g., a Web address); and if available, provide registration information, including the registration number.	16
**Eligibility criteria**	6	Specify characteristics of the sources of evidence used as eligibility criteria (e.g., years considered, language, and publication status), and provide a rationale.	13
**Information sources***	7	Describe all information sources in the search (e.g., databases with dates of coverage and contact with authors to identify additional sources), as well as the date the most recent search was executed.	4 & 21
**Search**	8	Present the full electronic search strategy for at least 1 database, including any limits used, such that it could be repeated.	21
**Selection of sources of evidence†**	9	State the process for selecting sources of evidence (i.e., screening and eligibility) included in the scoping review.	4
**Data charting process‡**	10	Describe the methods of charting data from the included sources of evidence (e.g., calibrated forms or forms that have been tested by the team before their use, and whether data charting was done independently or in duplicate) and any processes for obtaining and confirming data from investigators.	4 & 5
**Data items**	11	List and define all variables for which data were sought and any assumptions and simplifications made.	4 & 5
**Critical appraisal of individual sources of evidence§**	12	If done, provide a rationale for conducting a critical appraisal of included sources of evidence; describe the methods used and how this information was used in any data synthesis (if appropriate).	5
**Synthesis of results**	13	Describe the methods of handling and summarizing the data that were charted.	5

**RESULTS**			
**Selection of sources of evidence**	14	Give numbers of sources of evidence screened, assessed for eligibility, and included in the review, with reasons for exclusions at each stage, ideally using a flow diagram.	5
**Characteristics of sources of evidence**	15	For each source of evidence, present characteristics for which data were charted and provide the citations.	5 & 24
**Critical appraisal within sources of evidence**	16	If done, present data on critical appraisal of included sources of evidence (see item 12).	Not applicable
**Results of individual sources of evidence**	17	For each included source of evidence, present the relevant data that were charted that relate to the review questions and objectives.	5 to 14, and 24 onwards
**Synthesis of results**	18	Summarize and/or present the charting results as they relate to the review questions and objectives.	5 to 14
**DISCUSSION**			
**Summary of evidence**	19	Summarize the main results (including an overview of concepts, themes, and types of evidence available), link to the review questions and objectives, and consider the relevance to key groups.	14
**Limitations**	20	Discuss the limitations of the scoping review process.	15
**Conclusions**	21	Provide a general interpretation of the results with respect to the review questions and objectives, as well as potential implications and/or next steps.	15
**FUNDING**			
**Funding**	22	Describe sources of funding for the included sources of evidence, as well as sources of funding for the scoping review. Describe the role of the funders of the scoping review.	15

2 Supplementary material 2. Search strategy

I. Forward citation searching

Conducted in WoS and Scopus to identify articles that had cited at least one of the four seminal articles on candidacy [2-5]. We did not limit the search to articles that had cited the Dixon-Woods et al. 2006 paper and definition of candidacy, given the risks that it might have missed papers citing more recent applications of the Candidacy framework.

Database	Date conducted	Search string / filters applied	Results
WoS	21/09/2022	- Cite at least one of: Dixon-Woods et al. (2006)[2]; Mackenzie et al. (2011)[3]; Mackenzie et al. (2013)[4]; Mackenzie et al. (2015)[5].	977
Scopus	26/09/2022	- As above	1069
WoS	12/11/2024	- Cite at least one of: Dixon-Woods et al. (2006)[2]; Mackenzie et al. (2011)[3]; Mackenzie et al. (2013)[4]; Mackenzie et al. (2015)[5].<br>- Limit to citations from 2022 onwards	360
Scopus	12/11/2024	- As above	445

II. Related document search

Using the 'related documents' search function in Scopus and the 'related records' function in WoS to identify articles that shared references with Mackenzie et al. (2011) [3], Mackenzie et al. (2013) [4] or Mackenzie et al. (2015) [5] and which include the term "candida*" in any field.

Database	Date conducted	Search string / filters applied	Results
WoS	21/09/2022	- Includes the term "candida*" (in any field)<br>- Shares a reference with Mackenzie et al. (2011)[3]; Mackenzie et al. (2013)[4]; Mackenzie et al. (2015)[5].	314
Scopus	26/09/2022	- As above	739
WoS	12/11/2024	- Includes the term "candida*" (in any field)<br>- Shares a reference with Mackenzie et al. (2011)[3]; Mackenzie et al. (2013)[4]; Mackenzie et al. (2015)[5].<br>- Limit to citations from 2022 onwards	67
Scopus	12/11/2024	- As above	959

III. Supplementary search

Wider literature search to identify papers that use the term 'candidacy' in addition to healthcare and/or healthcare access related terms, that have not been captured in the previous searches.

Database	Date conducted	Search string / filters applied	Results
WoS	21/09/2022	- "(TI=(candidac*) OR AB=(candidac*)) AND (TI=(health OR medic* OR care OR healthcare OR surgery OR surgical OR treatment* OR service* OR access*) OR AB=(health OR medic* OR care OR healthcare OR surgery OR surgical OR treatment* OR service* OR access*))"	2810

		- Publication dates 2006-2022<br>- Indexes: SCI-EXPANDED, SSCI, A&HCI, CPCI-S, CPCI-SSH, BKCI-S, BKCI-SSH, ESCI, CCR-EXPANDED, IC.	
Scopus	26/09/2022	- “TITLE-ABS (candidac*) AND TITLE-ABS (health OR medic* OR care OR healthcare OR surgery OR surgical OR treatment* OR service* OR access*)<br>- Publication dates 2006-2022	3327
WoS	12/11/2024	- “(TI=(candidac*) OR AB=(candidac*)) AND (TI=(health OR medic* OR care OR healthcare OR surgery OR surgical OR treatment* OR service* OR access*) OR AB=(health OR medic* OR care OR healthcare OR surgery OR surgical OR treatment* OR service* OR access*))”<br>- Publication dates 01/09/2022-2025<br>- Indexes: SCI-EXPANDED, SSCI, A&HCI, CPCI-S, CPCI-SSH, BKCI-S, BKCI-SSH, ESCI, CCR-EXPANDED, IC.	514
Scopus	12/11/2024	- “TITLE-ABS (candidac*) AND TITLE-ABS (health OR medic* OR care OR healthcare OR surgery OR surgical OR treatment* OR service* OR access*)<br>- Publication dates 01/09/2022-2025	745

IV. Google Scholar search

A search for articles citing the original Dixon-Woods et al. (2006) paper [2], limited to those containing either the word “candidacy” or “candidacies”.

Database	Date conducted	Search string / filters applied	Results
Google Scholar	26/09/2022	- Articles citing Dixon-Woods et al. (2006)<br>- Articles containing “candidacy” or “candidacies”	269
Google Scholar	12/11/2024	- Articles citing Dixon-Woods et al. (2006)<br>- Articles containing “candidacy” or “candidacies”<br>- Since 2022	146

V. Additional thesis search

A further search using the Open Access Thesis and Dissertations platform on 4th October 2022.

Database	Date conducted	Search string / filters applied	Results
Open Access Thesis and Dissertations	04/10/2022	- abstract:(candidacy) AND abstract:("general practic*" OR "primary care" OR "family practic*" OR "family physician*" OR "family doctor*" OR "family medicine" OR GP*)<br>- Publication date limited to 2006 - 2022	11
Open Access Thesis and Dissertations	12/11/2024	- abstract:(candidacy) AND abstract:("general practic*" OR "primary care" OR "family practic*" OR "family physician*" OR "family doctor*" OR "family medicine" OR GP*)<br>- Publication date limited to 2022 - 2025	7

3 Supplementary material 3. Included studies

Authors	Type of paper	Research design and methods	Search	Geographic focus	Aim	Study population	Age range	Sample size	% female	Ethnicity or description of background
**Abbott et al. (2022) [6]**	Empirical	Qualitative interview study	Updated	Australia	To explore general practice care for people with a history of substance use disorders from the perspectives of women involved with the criminal justice system	Women in prisons who identified as having past or present substance use related health or social problems	19 to 59 years	39	100%	Aboriginal and Torres Strait Islanders: 17<br>Diverse backgrounds: 9<br>Others not given.
**Abbott et al. (2017) [7]**	Empirical	Qualitative interview study	Original	Australia	To examine how women in contact with the prison system experience access to health care, particularly those with histories of problematic substance misuse	Women in prisons, particularly those with histories of problematic substance misuse	19 to 59 years	40	100%	Aboriginal and Torres Strait Islanders: 17<br>Diverse backgrounds: 6<br>Others not given.
**Adams et al. (2024) [8]**	Empirical	Qualitative interview study	2nd update	UK	To explore how National Health Service (NHS) workers seek and access long covid healthcare	NHS workers experiencing long covid at NHS locales in Scotland	18 to 66+ years	50	84%	BAME: 6<br>Others not given.
**Albanese. (2023) [9]**	Thesis	Systematic review and qualitative interview study	2nd update	UK	To investigate asylum seekers', refugees', and undocumented migrants' mental health and psychosocial experiences in the postmigration context	Asylum seekers, refugees and undocumented migrants based in the Glasgow area.	All aged >18 years (9 young, 7 middle age, 2 older adults)	18 interviews	38%	Nigeria: 2<br>Pakistan: 2<br>Ethiopia: 1<br>Eritrea: 8<br>El Salvador: 2<br>Jordan: 2<br>Senegal: 1
**Annandale et al. (2007) [10]**	Review	Qualitative review and synthesis	Original	UK	To present a theoretically-grounded framework for understanding gender issues in access to healthcare	No specific group	Qualitative synthesis – sample sizes and demographics not reported.			
**Baggaley et al. (2023) [11]**	Empirical	Online survey and qualitative interviews	2nd update	UK	To explore the Cerebral Palsy diagnostic journey for community identified infants using the concept of candidacy	Caregivers of children with Cerebral Palsy, healthcare specialists in Cerebral Palsy	31 to 61 years	255 in survey<br>22 interviews	Survey: 96%<br>Patient interviewees: 41%	Survey:<br>White: 248<br>Asian :1<br>Black African: 1<br>Black Caribbean: 1<br><br>Interviews:<br>Majority were White European
**Bamidele et al. (2022)**	Review	Systematic review with	Updated	UK, USA, Canada	To synthesise published findings on barriers and	Black men who had undergone	49 to 85 years	139 patients in ten qualitative studies	0%	African American: 60

**[12]**		qualitative synthesis			facilitators to Black men accessing and utilising post-treatment psychosocial support after prostate cancer treatment	active treatment for prostate cancer				Black Caribbean: 60<br>Black African: 18<br>Unspecified: 1
**Bidmead et al. 2024 [13]**	Empirical	Qualitative study involving interviews and focus groups	2nd update	UK	To understand barriers to healthcare access faced by families living on low incomes to validate and support further development of a Poverty Proofing© healthcare tool	Parents on low-incomes and professionals working in the Voluntary, Community and Social Enterprise sector	Parents:<br>20 to 60 years<br><br>Professionals:<br>Not provided (all over 30)	31 (23 parents, 8 professionals)	96% (parents)<br>100% (professionals)	Not provided
**Black (2022) [14]**	Thesis	Qualitative study involving newspaper analysis, focus groups and individual interviews.	Updated	UK (mainly Scotland)	To identify and explore influences on how asylum seeking and refugee women access preventive healthcare, with a case study of cervical screening	Women who are asylum seekers and refugees, healthcare and community workers	28 to 46 years (patients)	26 (17 patients, 9 professionals)<br>198 newspaper articles	100%	Patients (numbers not provided):<br>Congo,<br>Iraq,<br>Guinea Bissau<br>Zimbabwe<br>Sudan<br>Libya<br>Algeria<br>Pakistan<br><br>Healthcare workers:<br>Pakistani: 1<br>White Scottish: 6
**Blane et al. (2020) [15]**	Review	Realist review	Original	23 papers from the USA, 5 from the UK and one each from Australia and Israel	To identify what works, and why, in the identification and referral of adults with comorbid obesity in primary care	Adults with obesity	N=30 papers reporting on 27 studies which included a total of 124872 participants. Few studies provided detailed information on age, gender or ethnicity.			
**Blane (2018) [16]**	Thesis	Mixed methods study including interviews, analysis of referrals, and a realist review	Updated	UK (mainly Scotland)	To examine the role of primary care in managing adults with co-morbid obesity	Adults with obesity, senior dieticians, primary care professionals	Interviews:<br>Not provided (senior dieticians)<br>24 to 74 years (patients)<br>30 to 60 years (primary care professionals)	Interviews: 9 senior dietitians, 20 patients, 17 primary care professionals.<br><br>Referrals analysed: 9,677<br><br>Review: 30 papers	Interviews:<br>78 (senior dieticians)<br>80% (patients)<br>76% (primary care professionals)<br><br>Referrals:	Not provided

							Referrals:<br>18 to 65+ years		71%	
**Booker et al. (2019) [17]**	Empirical	Ethnographic study	Original	UK	To explore what factors shape a service user's decision to call an emergency ambulance for a 'primary care sensitive' condition	Patients receiving ambulance treatment for a situation potentially manageable in primary care	18 to 92 years	50 cases	60%	Not provided
**Bristow et al. (2011) [18]**	Empirical	Qualitative interview study	Original	England	To understand the experience of seeking care for distress from the perspective of potential patients from "hard-to-reach" groups	People experiencing eating disorders, homelessness asylum seeking and from BME communities	18 to 81 years	34	71%	Chinese: 5<br>Irish: 4<br>Somali: 3<br>South Asian: 5
**Burch et al. (2019) [19]**	Empirical	Qualitative study involving interviews and focus groups	Original	England	To understand how clinicians are "diagnosing" older patients with pre-diabetes	Older patients with pre-diabetic blood parameters	Not provided	24	58%	Not provided
**Canvin et al. (2018) [20]**	Empirical	Qualitative interview study	Original	England and Wales	To explore how the literature overlooks 'healthy' and/or non-users of services, through older adults' accounts of seeking assistance	Older people aged 65+ years	68 to 95 years	40	50%	Not provided
**Chase et al. (2017) [21]**	Empirical	Qualitative interview study	Original	Canada	To elicit rich retrospective narratives from asylum seekers of difficulties in pursuit of healthcare during the period of contested "deservingness"	Asylum seekers	Not provided (average age 36.7 years)	26	54%	Sub-Saharan Africa: 10<br>North Africa: 3<br>Middle East: 3<br>South Asia: 2<br>Southeast Asia: 1<br>Caribbean: 5<br>South America: 1
**Chew-Graham et al. (2012) [22]**	Empirical	Secondary analysis of two qualitative data sets	Original	England	To explore why older people with depression may not present to primary care	Older people with depression	58 to 84 years	19	Not provided	South Asian: 1<br>African Caribbean: 2<br>Polish: 1<br>Caucasians: 15
**Dakin et al. (2024) [23]**	Empirical	Longitudinal study with ethnography, interviews, focus groups,	2nd update	UK	To understand how digital technologies, processes and pathways can lead to widening inequities of access, and	Patients and professionals in UK general practices	Not provided	174 interview participants, workshops involving over 200 people.	Not provided	Not provided

		stakeholder workshops, and demographic data			produce a novel theory of digital candidacy					
**Dando et al. (2019) [24]**	Empirical	Qualitative interview study	Original	England	To consider the experiences of Albanian female survivors of sex slavery who displayed significant and complex health needs, and their challenges to accessing healthcare	Albanian female survivors of sex slavery	21 to 29 years	7	100%	All were Albanian
**Dennis et al. (2015) [25]**	Empirical	Mixed methods study using surveys, interviews and community forums	Original	Australia	To determine the views of Aboriginal people on a brokerage model for access to community-based health services in an urban setting	Aboriginal people	Not provided (average age 50.2 years)	127 in survey (120 Aboriginal people, 7 professionals)<br>44 interviews (all Aboriginal people)	63.6% (Aboriginal people)	120 Aboriginal people Not provided for professionals.
**Depares & Culley (2022) [26]**	Empirical	Ethnographic Study	Updated	Malta	To explore the nature of healthcare encounters between asylum seekers and healthcare professionals in Maltese primary healthcare	Asylum seekers and healthcare professionals	25 to 54 years (asylum seekers)	22 interviews (11 asylum seekers, four cultural mediators and seven healthcare professionals), 130 observations hours	36% (asylum seekers)	Eritrea: 2<br>Somalia: 7<br>Nigeria: 2<br>Mali: 1<br>Sierra Leone: 1<br>Iraq: 1<br>Senegal: 1<br>Others not given
**Ellis et al. (2015) [27]**	Empirical	Qualitative interview study	Original	England	To explore how non-attenders of NHS Health Checks perceive the programme, to identify reasons for non-attendance and inform strategies to improve uptake	Non-attenders of NHS Health Checks	Not provided (average age 52.9 years)	41	54%	White British: 38<br>British Pakistani: 2<br>British Asian: 1
**Estupiñán et al. (2024) [28]**	Empirical	Secondary data analysis of survey data	2nd Update	UK	To describe and summarise patterns of inequities in breast cancer care	Secondary data analysis of the 2017/2018 English National Cancer Patient Experience Survey	16 to 75+ years	25408	100%	White British: 22729<br>Other White: 1082<br>Mixed ethnicity: 222<br>Asian: 806<br>Black: 437<br>Other ethnicity: 132
**Ford et al.**	Empirical	Qualitative study	Original	UK	To explore the barriers to	Socio-	67 to 87 years	15 (patient	60% (interviews)	Not provided

**(2018) [29]**		with interviews and focus groups			accessing primary care for socio-economically disadvantaged older people in rural areas	economically disadvantaged older people who live in rural areas and healthcare professionals	(interviews)	interviews)<br>16 (in 4 professional focus groups)		
**Garrett et al. (2012) [30]**	Empirical	Meta-ethnography	Original	UK	To develop an explanatory framework of the problems accessing primary care services experienced by British South Asian patients with a long-term condition or mental health problem	British South Asians with diabetes, coronary heart disease or depression	Review of 12 studies including 469 participants. Three included only female British South Asian participants. Four studies had mixed-ethnicity samples (British South Asian participants as focus of the study but with white-British and other Black and Minority Ethnic comparison groups) and eight had British South Asian only samples. Age range 16 to >90 years. Gender proportions not provided for all studies.			
**Goddard (2009) [31]**	Empirical	Policy case study	Original	England	To consider four example policies aimed at improving access and examine the evidence of their success	Not applicable	Not applicable	Not applicable	Not applicable	Not applicable
**Guassora et al. (2024) [32]**	Empirical	Qualitative interview study	2nd update	Denmark	To analyse the mechanisms at play in the adjudications made by professionals and socially vulnerable patients with type 2 diabetes about their eligibility for care	Patients with diabetes living in deprived areas and healthcare professionals	29 to 72 years (patients)<br>25 to 72 years (healthcare professionals)	24 (14 patients, 10 healthcare professionals)	29% (patients)<br>60% (healthcare professionals)	Not provided
**Herlitz et al. (2024) [33]**	Empirical	Qualitative study using podcasting, paired discussions and focus groups	2nd update	UK	To explore care-experienced young people's views and experiences of accessing general practice and dental services and attending health reviews in England	Care-experienced young people aged between 13 and 25 years	13 to 22 years	14	50%	White: 5<br>Mixed: 6<br>Black: 3
**Høj et al. (2019) [34]**	Empirical	Synthesis of access theories and framework development for access to Hepatitis C care	Original	No specific geographical focus	To synthesise and clarify the conceptual underpinnings of access to health care for people who inject drugs	People who inject drugs and are at risk of Hepatitis C	Not applicable	Not applicable	Not applicable	Not applicable
**Hudson et al. (2016) [35]**	Empirical	Qualitative interview study	Original	UK	To identify barriers to optimal care and good asthma control for South Asian children	South Asian Children with asthma, and their parents/carers	Not provided	49	Not provided	Indian Gujarati: 19<br>Pakistani: 7<br>Bangladeshi: 9

										Indian Punjabi: 14
**Hunter et al. (2013) [36]**	Empirical	Qualitative interview study	Original	England	To explore how patients with long-term conditions choose between available healthcare options during a health crisis	Patients with chronic obstructive pulmonary disease; coronary heart disease; asthma; or diabetes.	39 to 86 years	50	48%	White: 44<br>Black or Black British-Caribbean: 3<br>Black or Black British-African: 2<br>Mixed White and Black Caribbean: 1
**Huxley et al. (2015) [37]**	Review	Realist review	Original	UK	To assess the potential impact of the availability of digital clinician–patient communication on marginalised groups' access to general practice	People with mental illness, refugees, asylum seekers, homeless people, Travellers, and carers	Review of 70 papers with no details on age, gender or ethnicity of participants in included studies provided.			
**Ip et al. (2022) [38]**	Empirical	Qualitative interview study	Updated	UK	To understand how patients' help-seeking experiences in primary care for colorectal cancer symptoms during COVID-19 were affected by their socioeconomic status	People who contacted primary care about symptoms related to colorectal cancer during COVID-19 pandemic	25 to 78 years	39	51%	White: 28<br>Indian: 2<br>Black British: 4<br>Arab: 1<br>Chinese: 1<br>Asian/Asian British: 1<br>Mixed: 2
**Ip et al. (2022) [39]**	Empirical	Qualitative interview study	Updated	UK	To explore healthcare professional and patient perceptions of the main changes to colorectal cancer delivery during the COVID-19 pandemic and how they impacted on socioeconomic inequalities in care	People with symptoms of colorectal cancer and healthcare professionals	Not provided (average age of patients 57 years)	45 (15 patients, 30 healthcare professionals)	60% (patients)<br>60% (healthcare professionals)	Patients:<br>White: 12<br>Black: 1<br>Indian: 1<br>Asian/Asian British: 1<br><br>Healthcare professionals:<br>White: 24<br>Chinese: 2<br>Mixed: 1<br>Indian: 3
**Isaacs (2018) [40]**	Thesis	Ethnography including community engagement, focus groups and interviews	Updated	UK	To understand the health-related experiences of accessing primary and preventive health services for asylum seekers and	Asylum seekers and refugees from Sub Saharan Africa and healthcare professionals	20 to 60+ years (asylum seekers)	39 (27 asylum seekers, 12 healthcare professionals)	58% (asylum seekers)	Eritrea: 8<br>Malawi: 2<br>Zambia: 1<br>Zimbabwe: 9<br>Ghana: 2<br>Sudan:1

					refugees					South Africa: 1<br><br>Healthcare professionals: not provided.
**Jouanny et al. (2024) [41]**	Review	Systematic literature review using mixed methods	2nd update	No specific geographical focus	To review the barriers and facilitators to help-seeking among women with stigmatised pelvic health symptoms	Women experiencing symptoms pelvic health symptoms	Review of 86 articles, representing over 20,000 women from 24 high income countries. 36% of papers were from Europe, 31% from North America, 21% from East Asia and Pacific, 7% from Middle East and North Africa, 2.33% Worldwide, and 1.16% from Latin America. No other details on age, gender or ethnicity of participants in included studies provided.			
**Khan & Kobayashi (2015) [42]**	Review	Scoping literature review	Original	Canada but includes articles from UK, USA and Australia	To identify the barriers to uptake and utilization of health promotion interventions among ethnocultural minority older adults	Older adults from ethnocultural minority groups	Review of 25 documents with an emphasis on older ethnocultural minorities in Canada, UK, USA and Australia. No other details on age, gender or ethnicity of participants in included studies provided.			
**Koehn (2009) [43]**	Empirical	Qualitative study using focus groups, participant observation, interviews and workshops	Original	Canada	To examine barriers that Vietnamese, Hispanic and Punjabi seniors experience in accessing Home and Community Care	Vietnamese, Hispanic and Punjabi older people, and healthcare and multi-cultural service providers	Not provided (focused on 'seniors')	82 (56 older people, 26 health care and multicultural service providers)	Not provided	Punjabi, Vietnamese and Hispanic communities
**Koehn et al. (2016) [44]**	Empirical	Qualitative interview study	Original	Canada	To understand the challenges to securing a diagnosis of dementia, especially among ethnic minority groups, and consider the policy and practice implications	People with dementia from ethnic minority groups and their family caregivers	Not provided	63	52%	Anglo-Canadians in Calgary: 13<br>Francophone Canadians in Ottawa: 14<br>Indo-Canadians in Toronto: 15<br>Chinese Canadians in Vancouver: 21
**Koehn et al. (2024) [45]**	Review	Systematic review (using Critical Interpretative Synthesis)	2nd update.	Of relevance to a North American context (broadly defined to include studies outside North America if relevant)	To explore the experiences of individuals living with rheumatoid arthritis and their formal care providers	Adults with rheumatoid arthritis	Review of 110 papers with no summary of age distributions, gender or ethnicity of participants in included studies provided.			
**Lane & Vatanparast (2022) [46]**	Empirical	Qualitative interview study	Updated	Canada	To explore newcomer families' experiences with healthcare during their	Newcomers to Canada in the previous 5 years	Not provided	44 (22 parents, 22 service providers)	68% (parents only)	Parents:<br>Eastern Europe: 1

					first 5 years and their suggestions for improvements to inform development of an accessible newcomer healthcare model	(immigrant population) and service providers				Latin America: 1<br>Africa: 2<br>US: 2<br>Western Europe: 2<br>Asia: 6<br>Middle East: 8
**Lien (2021) [47]**	Empirical		Updated	Norway	To present attitudes of providers and receivers of healthcare and see how health workers and Sub-Saharan African women view equal access for women from countries practicing Female Genital Mutilation/Cutting	Sub-Saharan African women and healthcare providers	22 to 68 years (patients)<br>45 to 50 years (healthcare workers)	126 (55 women, 71 healthcare workers)	Patients were all female. Healthcare professionals were 'mostly women'	Patients:<br>Gambian: 20<br>Somalian: 20<br>Eritrean: 15
**Lindsay et al. (2024) [48]**	Review	Realist review	2nd update	UK	To address knowledge gaps around multiple missed appointments in UK primary care	People exhibiting a repeated tendency to not take up offers of health care	Literature review of 197 documents with no details on age, gender or ethnicity of participants in included studies provided. Studies conducted in UK, USA, Australia, Canada, Denmark, Germany, Israel, Malaysia, New Zealand, Switzerland			
**Llanwarne et al. (2017) [49]**	Empirical	Qualitative interview study	Original	England	To investigate patient accounts of negotiating service use, and the voiced notion of 'wasting the doctor's time' in general practice	Patients from practices scoring in the bottom 25% in the GP Practice Survey	19 and 96 years.	52	67%	White British: 45<br>White other: 3<br>Black: 3<br>Asian: 1
**Macdonald et al. (2016) [50]**	Empirical	Secondary analysis of three qualitative datasets	Original	Scotland	To propose a further dimension - illness identity - that extends the Candidacy framework	Patients with colorectal cancer or heart failure	48 to 86 years	30	50%	Not provided
**Machin et al. (2017) [51]**	Empirical	Qualitative interview study	Original	UK	To explore patients' perspectives of anxiety and depression in Rheumatoid Arthritis and preferences for disclosure and management of mood problems	People with rheumatoid arthritis and anxiety/ depression	40 to 70+ years	14	86%	All White British
**Mackenzie et al. (2019) [52]**	Empirical	Qualitative interview study (as part of larger study)	Original	UK	To explore discourse from women who have experienced domestic abuse about interacting with family doctors	Women who have experienced domestic abuse	20 to 70 years	20	100	All White British

**MacKichan et al. (2017) [53]**	Empirical	Ethnography involving observations, informal and formal interviewing	Original	England	To describe how processes of primary care access influence decisions to seek help at the emergency department	Patients with recent emergency department use (or a carer if aged <16), and healthcare professionals	23 to 72 years (patients/carers)	48 (19 staff, 29 patients/carers)	76% (patients/carers only)	Not provided
**Maclean et al. (2023) [54]**	Empirical	Qualitative interview study	2nd update	UK, USA, Netherlands, Australia, Canada	To explore people's accounts of establishing their need and experiences of healthcare for long covid symptoms	People with long covid	20 to 79 years	73	80%	Heterogenous categories meant summarisation of ethnicity was not possible
**Mastrocola et al. (2015) [55]**	Empirical	Qualitative interview study	Original	England	To explore the perspectives of women involved in street-based prostitution about access to health care for long-term conditions	Women involved in street-based prostitution	22 to 60 years	16	100%	White British: 94%
**Mawson (2022) [56]**	Thesis	Qualitative study involving qualitative evidence synthesis and interviews	Updated	UK with review including data from Australia, Canada, England, Ireland, Norway, Netherlands, New Zealand, Scotland	To explore barriers and facilitators to accessing Sexual and Reproductive Health in general practice, using candidacy to explore practitioner and public perspectives	People from deprived communities and black or ethnic minority groups and GPs and practice nurses who serve deprived populations	21 to 50 years (interviews)	20 (interviews)<br>37 studies (review)	65% (interviews)	Interviews:<br>Caucasian: 17<br>Chinese: 2<br>Asian: 1
**Methley (2017) et al. [57]**	Empirical	Qualitative interview study	Original	England	To explore perspectives and experiences of patients with multiple sclerosis and healthcare professionals of UK healthcare services	Patients with multiple sclerosis and healthcare professionals	18 to 80 years (patients)<br>21 to 60 years (healthcare professionals)	58 (24 patients, 34 healthcare professionals)	79% (patients)<br>82% (healthcare professionals)	Patients<br>White British: 23<br>Iranian: 1<br><br>Healthcare professionals<br>White British: 29<br>British Asian: 2<br>British Chinese: 1<br>Arab: 1<br>Other: 1<br>Mixed 1
**Methley et al. (2017) [58]**	Empirical	Qualitative interview study	Original	England	To explore perspectives and experiences of mental health support for	Patients with multiple sclerosis and healthcare	See above Methley (2017) et al. [57]	See above Methley (2017) et al. [57]	See above Methley (2017) et al. [57]	See above Methley (2017) et al. [57]

					people with multiple sclerosis and healthcare professionals	professionals				
**Methley (2015) [59]**	Thesis		Updated	UK	To explore the experiences of people with multiple sclerosis and professionals in the management of physical and psychological symptoms	Patients with multiple sclerosis and healthcare professionals	18 to 80 years (patients)<br>21 to 60 years (healthcare professionals)	59 (25 patient participants, 34 healthcare professionals)	80% (patients)<br>82% (healthcare professionals)	Patients:<br>White British: 24<br>Iranian: 1<br><br>Healthcare professionals:<br>White British: 29<br>British Asian: 2<br>British Chinese: 1<br>Arab: 1<br>Other: 1<br>Mixed: 1
**Mughal et al. (2021) [60]**	Empirical	Qualitative interview study	Updated	England	To explore the help-seeking behaviours, experiences of GP care, and access to general practice of young people who self-harm	Young people (aged 16-25 yrs) who self-harm	19 to 25 years	13	92%	White British: 7<br>Mixed: 3<br>Asian British: 1<br>White American: 1<br>Not disclosed: 1
**Novek & Menec (2021) [61]**	Empirical	Qualitative interview study	Original	Canada	To examine the process of accessing and delivering a diagnosis from perspectives of people living with young onset dementia, family, and providers	People living with young onset dementia and family members and care providers	57 to 66 years (people living with dementia)<br>20 to 76 years (family members)	36 (6 people living with dementia, 14 family members, 16 providers)	33% of people living with dementia<br>50% of family members	White Canadian: 20<br>(People living with dementia and family members)
**O'Brien et al. (2019) [62]**	Empirical	Qualitative interview study	Original	Australia	To explore patient factors that impact on the decision to progress to total knee replacement	Patients with knee osteoarthritis who were on a waiting list to undergo total knee replacement	52 to 80 years	27	48%	Not provided
**Osborn et al. (2024) [63]**	Empirical	Qualitative interview study	2nd Update	UK	To develop a better understanding of students' access to mental health services	Healthcare professionals working in university counselling services, general practice, and psychological services	27 to 75 years	23	57%	Not provided
**Peiris et al. (2012) [64]**	Empirical	Qualitative study involving focus	Original	Australia	To explore staff perspectives on health	Healthcare staff from Aboriginal	Not provided	37	Not provided	Not provided

		groups			systems issues that impact on access to optimal primary, specialist and hospital care and organisational barriers and enablers to improved quality of care	Medical Services				
**Pétrin et al. (2021) [65]**	Empirical	Qualitative study involving focus groups and interviews	Updated	Canada	To align the experiences of persons with multiple sclerosis in accessing healthcare services with the stages of candidacy	People with multiple sclerosis	Not provided (average age 49.6 years)	48	66%	Caucasian: 45
**Rijken et al. (2022) [66]**	Empirical	Questionnaire validation	2nd Update	Netherlands	To assess the internal and construct validity of the Dutch P3CEQ to capture the experience of person-centred coordinated care of people with chronic conditions	People with chronic conditions on a nationwide panel study in the Netherlands	Not provided (average age 67.9 years)	1098	54%	10 participants were of non-western origin
**Sinnott et al. (2024) [67]**	Review	Literature review (guided by Critical Interpretative Synthesis)	2nd Update	UK	To characterise how the Candidacy Framework can explain access to general practice	Patients seeking or eligible to seek care from general practice	Literature review of 229 articles with no details on age, gender or ethnicity of participants in included studies provided			
**Smyth et al. (2024) [68]**	Empirical	Qualitative interview study	2nd Update	UK	To understand the lived experiences of long COVID in people from ethnic minority groups	People from ethnic minority groups with long COVID	20 to 60+ years	31	52%	Arab: 3<br>Black: 10<br>South Asian: 10<br>Mixed heritage: 6<br>Other: 2
**Sundaresan et al. (2016) [69]**	Review	Literature review	Original	Australia but includes literature from other countries	To develop a conceptual framework for the consideration of 'radiation therapy access'	Patients with cancer	Literature review with no details on age, gender or ethnicity of participants or the number of included studies provided.			
**Tarrant et al. (2015) [70]**	Empirical	Qualitative study: Secondary analysis of qualitative interviews	Original	England	To explore patients' experiences of discontinuities in care and gain insight into how gaps come to be bridged or remain unresolved	Patients from general practice, a walk-in centre and community settings	13 to 80+ years	50	62%	White British: 42<br>Other ethnicity: 7
**Thomas et al. (2019) [71]**	Review	Literature review (systematic review and realist synthesis)	Original	10 studies from US and one from Australia	To identify whether a health service broker working with service providers can (a) identify individuals experiencing	Individuals experiencing vulnerability or disadvantage	Literature review of 11 articles with no details on age, gender or ethnicity of participants in included studies provided.			

					vulnerability who may benefit from improved access and (b) link these individuals with an appropriate primary care provider				
**Tookey et al. (2018) [72]**	Empirical	Qualitative study: Secondary analysis of qualitative interviews	Original	England	To capture participants' experiences of ongoing cancer symptoms and to explore help-seeking decisions	People over 50 experiencing 'cancer alarm symptoms'	No age range given (Aged ≥50 years)	62	47%	Not provided
**Turk et al. (2024) [73]**	Empirical	Qualitative interview study	2nd update.	UK	To identify facilitators and barriers to healthcare access for people with Long Covid	Adults with lived experience of Long Covid and healthcare professionals	20 to 59 years (patients)<br>20 to 69 years (healthcare professionals)	16 (8 patients, 8 healthcare professionals)	38% (patients)<br>63% (healthcare professionals	Patients:<br>White:6<br>Other ethnicity: 1<br>Asian/Asian British: 1<br><br>Healthcare professionals<br>White: 4<br>Black: 1<br>Mixed: 1<br>Missing: 2
**Westlake et al. 2022 [74]**	Empirical (secondary analysis)	Qualitative study: Secondary analysis of qualitative interviews	2nd Update	UK	To examine why and in what circumstances patients become more involved in their care and how professionals can enhance participation	Patients and healthcare professionals across Southwest England	Secondary analysis of 110 interviews, 24 observations of practices, 8 observations of multidisciplinary meetings, 7 observations of peer-to-peer group meetings, and 7 focus groups. Age, gender and ethnicity data not provided.			
**Whitford et al. (2024) [75]**	Empirical	Qualitative interview study	2nd update	Australia	To identify barriers and enablers to sexual healthcare among young Aboriginal and Torres Strait Islander people in regional settings.	Young Aboriginal and Torres Strait Islander people	15 to 29 years	65	54%	All were Aboriginal and Torres Strait Islanders
**Williamson et al. (2023) [76]**	Empirical	Qualitative interview study	2nd update	UK	To explore experiences of accessing community and emergency care, from the perspectives of people with dementia, current and bereaved family caregivers	People with dementia living at home, their carers and bereaved carers	50 to 90+ years	35 (10 people with dementia, 11 current caregivers and 16 bereaved caregivers; 2 dyad, 33 individual interviews)	69%	White: 32<br>Black, Asian & Minority: 3
**Wilson et al. (2022) [77]**	Empirical	Two online surveys	Updated	UK	To profile provision and use of NHS and non-NHS services by people	People with fibromyalgia and healthcare	Not provided	2250 survey responses (549 patients, 1701	Not provided	Not provided

					with fibromyalgia	professionals		healthcare professionals)		
**Worthing et al. (2022) [78]**	Empirical	Qualitative study involving interviews and focus groups	Identified after searches	UK	To understand the processes and influences on refusal of registration for those without documentation	GP staff involved in registering new patients	Not provided	33 (13 individual, 20 focus group)	Not provided	Not provided

4 Supplementary material 4. Features of the Candidacy Framework used by included studies

**Authors**	**Elements of the Candidacy Framework used**							**Does the paper critique or modify the Candidacy Framework**
	Identification	Navigation	Permeability	Appearances	Adjudications	Offers and Resistance	Operating Conditions	
**Abbott et al. (2022) [6]**	Yes	Yes	Yes	Yes	Yes	Yes	Yes	No
**Abbott et al. (2017) [7]**	Yes	Yes	Yes	Yes	Yes	Yes	Yes	No
**Adams et al. (2024) [8]**	Yes	Yes	Yes	Yes	Yes	Yes	Yes	Yes
**Albanese. 2023 [9]**	Yes	Yes	Yes	Yes	Yes	Yes	Yes	Yes
**Annandale et al. (2007) [10]**	Yes	Yes	Yes	Yes	Yes	No	Yes	Yes
**Baggaley et al. (2023) [11]**	Yes	Yes	Yes	Yes	Yes	No	Yes	No
**Bamidele et al. (2022) [12]**	Yes	Yes	Yes	Yes	Yes	Yes	Yes	No
**Bidmead et al. 2024 [13]**	Yes	Yes	Yes	Yes	Yes	No	Yes	No
**Black (2022) [14]**	Yes	Yes	Yes	Yes	Yes	Yes	Yes	Yes
**Blane et al. (2020) [15]**	Yes	Yes	Yes	Yes	Yes	Yes	Yes	Yes
**Blane (2018) [16]**	Yes	Yes	Yes	Yes	Yes	Yes	Yes	Yes
**Booker et al. (2019) [17]**	No	No	No	No	No	No	No	No
**Bristow et al. (2011) [18]**	Yes	Yes	No	Yes	Yes	Yes	No	No

**Burch et al. (2019) [19]**	Yes	Yes	No	No	Yes	Yes	Yes	No
**Canvin et al. (2018) [20]**	Yes	No	No	No	No	Yes	No	Yes
**Chase et al. (2017) [21]**	Yes	Yes	Yes	Yes	Yes	Yes	Yes	Yes
**Chew-Graham et al. (2012) [22]**	Yes	Yes	No	No	No	Yes	No	No
**Dakin et al. (2024) [23]**	Yes	Yes	Yes	Yes	Yes	No	Yes	Yes
**Dando et al. (2019) [24]**	Yes	Yes	Yes	Yes	Yes	No	No	No
**Dennis et al. (2015) [25]**	No	Yes	Yes	Yes	No	Yes	No	No
**Depares & Culley (2022) [26]**	No	Yes	No	No	No	No	No	No
**Ellis et al. (2015) [27]**	No	No	No	No	No	No	No	No
**Estupiñán et al. (2024) [28]**	Yes	Yes	Yes	Yes	Yes	Yes	Yes	Yes
**Ford et al. (2018) [29]**	No	No	Yes	No	No	No	No	No
**Garrett et al. (2012) [30]**	Yes	Yes	No	Yes	Yes	Yes	No	Yes
**Goddard (2009) [31]**	No	Yes	Yes	No	Yes	Yes	No	Yes
**Guassora et al. (2024) [32]**	No	No	No	No	Yes	Yes	Yes	No
**Herlitz et al. (2024) [33]**	Yes	Yes	No	Yes	Yes	Yes	Yes	Yes
**Høj et al. (2019) [34]**	Yes	Yes	Yes	Yes	Yes	Yes	Yes	Yes
**Hudson et al. (2016) [35]**	Yes	Yes	Yes	Yes	Yes	Yes	Yes	No

**Hunter et al. (2013) [36]**	No	No	Yes	No	No	No	No	Yes
**Huxley et al. (2015) [37]**	No	No	No	No	No	No	No	No
**Ip et al. (2022) [38]**	No	Yes	No	Yes	No	Yes	No	No
**Ip et al. (2022) [39]**	No	Yes	Yes	Yes	Yes	No	No	No
**Isaacs (2018) [40]**	Yes	Yes	Yes	Yes	Yes	Yes	Yes	Yes
**Jouanny et al. (2024) [41]**	Yes	No	No	No	Yes	No	No	No
**Khan & Kobayashi (2015) [42]**	Yes	Yes	Yes	Yes	Yes	Yes	Yes	No
**Koehn (2009) [43]**	Yes	Yes	Yes	Yes	Yes	Yes	Yes	No
**Koehn et al. (2016) [44]**	Yes	Yes	Yes	Yes	Yes	Yes	Yes	No
**Koehn et al. (2024) [45]**	Yes	Yes	Yes	Yes	Yes	Yes	Yes	Yes
**Lane & Vatanparast (2022) [46]**	No	No	No	No	No	No	No	No
**Lien (2021) [47]**	Yes	Yes	Yes	Yes	Yes	No	No	Yes
**Lindsay et al. (2024) [48]**	Yes	Yes	Yes	No	Yes	Yes	Yes	No
**Llanwarne et al. (2017) [49]**	No	Yes	Yes	Yes	Yes	Yes	Yes	No
**Macdonald et al. (2016) [50]**	Yes	Yes	Yes	Yes	Yes	No	Yes	Yes
**Machin et al. (2017) [51]**	No	No	No	No	No	No	No	No
**Mackenzie et al. (2019)**	Yes	No	No	Yes	Yes	Yes	No	Yes

**[52]**								
**MacKichan et al. 2017) [53]**	Yes	Yes	No	No	No	No	No	No
**Maclean et al. (2023) [54]**	Yes	Yes	Yes	No	Yes	No	No	Yes
**Mastrocola et al. (2015) [55]**	Yes	Yes	Yes	Yes	No	No	No	No
**Mawson (2022) [56]**	Yes	Yes	Yes	Yes	Yes	Yes	Yes	Yes
**Methley (2017) et al. [57]**	Yes	Yes	Yes	Yes	Yes	Yes	Yes	No
**Methley et al. (2017) [58]**	Yes	No	Yes	No	No	No	Yes	No
**Methley (2015) [59]**	Yes	Yes	Yes	Yes	Yes	Yes	Yes	Yes
**Mughal et al. (2021) [60]**	Yes	Yes	No	Yes	Yes	No	No	No
**Novek & Menec (2021) [61]**	Yes	Yes	Yes	Yes	Yes	Yes	Yes	Yes
**O'Brien et al. (2019) [62]**	Yes	Yes	Yes	Yes	Yes	Yes	Yes	Yes
**Osborn et al. (2024) [63]**	Yes	Yes	Yes	Yes	Yes	Yes	Yes	Yes
**Peiris et al. (2012) [64]**	No	Yes	No	Yes	No	No	No	Yes
**Pétrin et al. (2021) [65]**	Yes	Yes	Yes	Yes	Yes	Yes	Yes	Yes
**Rijken et al. (2022) [66]**	No	No	No	No	No	No	No	No
**Sinnott et al. (2024) [67]**	Yes	Yes	Yes	Yes	Yes	Yes	Yes	Yes
**Smyth et al. (2024) [68]**	No	Yes	No	No	No	No	No	No

**Sundaresan et al. (2016) [69]**	No	Yes	Yes	No	No	No	No	No
**Tarrant et al. (2015) [70]**	Yes	Yes	Yes	Yes	Yes	No	No	No
**Thomas et al. (2019) [71]**	Yes	Yes	Yes	Yes	No	Yes	No	Yes
**Tookey et al. (2018) [72]**	No	No	No	Yes	Yes	Yes	Yes	Yes
**Turk et al. (2024) [73]**	Yes	Yes	Yes	Yes	No	No	No	No
**Westlake et al. 2022 [74]**	No	No	No	No	No	Yes	No	No
**Whitford et al. (2024) [75]**	Yes	Yes	Yes	Yes	Yes	Yes	Yes	No
**Williamson et al. (2023) [76]**	Yes	Yes	No	No	No	No	Yes	Yes
**Wilson et al. (2022) [77]**	No	No	Yes	Yes	Yes	No	Yes	No
**Worthing et al. (2022) [78]**	No	No	No	No	Yes	No	No	No

5 References

1. Tricco, A.C., et al., *PRISMA Extension for Scoping Reviews (PRISMA-ScR): Checklist and Explanation.* Ann Intern Med, 2018. **169**(7): p. 467-473.
2. Dixon-Woods, M., et al., *Conducting a critical interpretive synthesis of the literature on access to healthcare by vulnerable groups.* BMC Med Res Methodol, 2006. **6**: p. 35.
3. Mackenzie, M., et al., *What is the 'problem' that outreach work seeks to address and how might it be tackled? Seeking theory in a primary health prevention programme.* BMC Health Serv Res, 2011. **11**: p. 350.
4. Mackenzie, M., et al., *Is 'Candidacy' a Useful Concept for Understanding Journeys through Public Services? A Critical Interpretive Literature Synthesis.* Social Policy & Administration, 2013. **47**(7): p. 806-825.
5. Mackenzie, M., et al., *Intersections and Multiple 'Candidacies': Exploring Connections between Two Theoretical Perspectives on Domestic Abuse and Their Implications for Practicing Policy.* Social Policy and Society, 2015. **14**(1): p. 43-62.
6. Abbott, P., et al., *Welcomeness for people with substance use disorders to general practice: a qualitative study.* FAMILY PRACTICE, 2022. **39**(2).
7. Abbott, P., et al., *Medical homelessness and candidacy: Women transiting between prison and community health care.* International Journal for Equity in Health, 2017. **16**(1).
8. Adams, N.N., et al., *Disrupted Candidacy: A Longitudinal Examination of the Constrained Healthcare-Access Journeys of National Health Service Workers in Scotland Seeking Supports for Long COVID Illness.* Health Expectations, 2024. **27**(5).
9. Albanese, A., *Exploring the mental health and psychosocial experiences of asylum seekers, refugees and undocumented migrants in the post-migration context.* 2023, University of Glasgow.
10. Annandale, E., et al., *Gender and access to healthcare in the UK: A critical interpretive synthesis of the literature.* Evidence and Policy, 2007. **3**(4): p. 463-486.
11. Baggaley, J.J.E., et al., *From Eligibility to Diagnosis: Candidacy and the Complex Journey of Cerebral Palsy Diagnosis Within Primary Care.* 2023.
12. Bamidele, O.O., et al., *Barriers and facilitators to accessing and utilising post-treatment psychosocial support by Black men treated for prostate cancer—a systematic review and qualitative synthesis.* Supportive Care in Cancer, 2022. **30**(5).
13. Bidmead, E., et al., *Poverty proofing healthcare: A qualitative study of barriers to accessing healthcare for low-income families with children in northern England.* Plos one, 2024. **19**(4): p. e0292983.
14. Black, A., *An exploration of the factors that influence how asylum seeking or refugee women access preventive healthcare, using cervical screening as a case study.* 2022, University of Glasgow.
15. Blane, D.N., S. Macdonald, and C.A. O'Donnell, *What works and why in the identification and referral of adults with comorbid obesity in primary care: A realist review.* Obesity Reviews, 2020. **21**(4).
16. Blane, D.N., *Understanding the role of primary care in the management of adults with co-morbid obesity : a mixed methods programme.* 2018, University of Glasgow.
17. Booker, M.J., et al., *Ambulance use for 'primary care' problems: An ethnographic study of seeking and providing help in a UK ambulance service.* BMJ Open, 2019. **9**(10).
18. Bristow, K., et al., *Help seeking and access to primary care for people from "hard-to-reach" groups with common mental health problems.* International journal of family medicine, 2011. **2011**.
19. Burch, P., et al., *Understanding the diagnosis of pre-diabetes in patients aged over 85 in English primary care: A qualitative study.* BMC Family Practice, 2019. **20**(1).
20. Canvin, K., et al., *Seeking assistance in later life: How do older people evaluate their need for assistance?* Age and Ageing, 2018. **47**(3): p. 466-473.
21. Chase, L.E., et al., *The gap between entitlement and access to healthcare: An analysis of "candidacy" in the help-seeking trajectories of asylum seekers in Montreal.* Social Science and Medicine, 2017. **182**: p. 52-59.

22. Chew-Graham, C., et al., *Why may older people with depression not present to primary care? Messages from secondary analysis of qualitative data.* Health and Social Care in the Community, 2012. **20**(1): p. 52-60.
23. Dakin, F.H., et al., *Access and triage in contemporary general practice: A novel theory of digital candidacy.* Social Science & Medicine, 2024. **349**.
24. Dando, C.J., et al., *Health inequalities and health equity challenges for victims of modern slavery.* Journal of Public Health, 2019. **41**(4): p. 681-688.
25. Dennis, S., et al., *Experiences and views of a brokerage model for primary care for Aboriginal people.* Australian Health Review, 2015. **39**(1): p. 26-32.
26. Depares, J. and L. Culley, *Healthcare encounters between asylum seekers and health professionals in Maltese primary care.* Journal of Research in Nursing, 2022. **27**(3).
27. Ellis, N., et al., *A qualitative investigation of non-response in NHS health checks.* Archives of Public Health, 2015. **73**(1).
28. Estupiñán Fdez. de Mesa, M., et al., *Using the Candidacy Framework to understand individual, interpersonal, and system level factors driving inequities in women with breast cancer: a cross-sectional study.* BJC Reports, 2024. **2**(1): p. 83.
29. Ford, J.A., et al., *Access to primary care for socio-economically disadvantaged older people in rural areas: A qualitative study.* PLoS ONE, 2018. **13**(3).
30. Garrett, C.R., et al., *Accessing primary health care: A meta-ethnography of the experiences of British South Asian patients with diabetes, coronary heart disease or a mental health problem.* Chronic Illness, 2012. **8**(2): p. 135-155.
31. Goddard, M., *Access to health care services - An English policy perspective.* Health Economics, Policy and Law, 2009. **4**(2): p. 195-208.
32. Guassora, A.D., et al., *Adjudications and tinkering with care for socially vulnerable patients with type 2 diabetes in general practice.* Scandinavian Journal of Primary Health Care, 2024. **42**(2): p. 295-303.
33. Herlitz, L., et al., *Care-experienced young people's views and experiences of accessing general practice and dental services and attending health reviews in England: a qualitative study.* Bmc Primary Care, 2024. **25**(1).
34. Høj, S.B., et al., *Conceptualising access in the direct-acting antiviral era: An integrated framework to inform research and practice in HCV care for people who inject drugs.* International Journal of Drug Policy, 2019. **72**: p. 11-23.
35. Hudson, N., et al., *Asthma management in British South Asian children: An application of the candidacy framework to a qualitative understanding of barriers to effective and accessible asthma care.* BMC Public Health, 2016. **16**(1).
36. Hunter, C., et al., *A qualitative study of patient choices in using emergency health care for long-term conditions: The importance of candidacy and recursivity.* Patient Education and Counseling, 2013. **93**(2): p. 335-341.
37. Huxley, C.J., et al., *Digital communication between clinician and patient and the impact on marginalised groups: A realist review in general practice.* British Journal of General Practice, 2015. **65**(641): p. e813-e821.
38. Ip, A., et al., *Socioeconomic differences in help seeking for colorectal cancer symptoms during COVID-19: a UK-wide qualitative interview study of patient experiences in primary care.* British Journal of General Practice, 2022. **72**(720).
39. Ip, A., et al., *Healthcare Professional and Patient Perceptions of Changes in Colorectal Cancer Care Delivery During the COVID-19 Pandemic and Impact on Health Inequalities.* Cancer Control, 2022. **29**.
40. Isaacs, A., *Keeping healthy and accessing primary and preventive health services in Glasgow : the experiences of refugees and asylum seekers from Sub Saharan Africa.* 2018, University of Glasgow.
41. Jouanny, C., P. Abhyankar, and M. Maxwell, *A mixed methods systematic literature review of barriers and facilitators to help-seeking among women with stigmatised pelvic health symptoms.* Bmc Womens Health, 2024. **24**(1).
42. Khan, M.M. and K. Kobayashi, *Optimizing health promotion among ethnocultural minority older adults (EMOA).* International Journal of Migration, Health and Social Care, 2015. **11**(4): p. 268-281.

43. Koehn, S., *Negotiating candidacy: Ethnic minority seniors' access to care.* Ageing and Society, 2009. **29**(4): p. 585-608.
44. Koehn, S., et al., *Negotiating access to a diagnosis of dementia: Implications for policies in health and social care.* Dementia, 2016. **15**(6): p. 1436-1456.
45. Koehn, S., et al., *Candidacy 2.0 (CC) - an enhanced theory of access to healthcare for chronic conditions: lessons from a critical interpretive synthesis on access to rheumatoid arthritis care.* Bmc Health Services Research, 2024. **24**(1).
46. Lane, G. and H. Vatanparast, *Adjusting the Canadian Healthcare System to Meet Newcomer Needs.* International Journal of Environmental Research and Public Health, 2022. **19**(7).
47. Lien, I.L., *Health workers and Sub Saharan African women's understanding of equal access to healthcare in Norway.* PLoS ONE, 2021. **16**(9 September).
48. Lindsay, C., et al., *Understanding the causes of missingness in primary care: a realist review.* Bmc Medicine, 2024. **22**(1).
49. Llanwarne, N., et al., *Wasting the doctor's time? A video-elicitation interview study with patients in primary care.* Social Science and Medicine, 2017. **176**: p. 113-122.
50. Macdonald, S., et al., *Illness identity as an important component of candidacy: Contrasting experiences of help-seeking and access to care in cancer and heart disease.* Social Science and Medicine, 2016. **168**: p. 101-110.
51. Machin, A., et al., *Improving recognition of anxiety and depression in rheumatoid arthritis: A qualitative study in a community clinic.* British Journal of General Practice, 2017. **67**(661): p. e531-e537.
52. Mackenzie, M., et al., *'You certainly don't go back to the doctor once you've been told, "I'll never understand women like you."' Seeking candidacy and structural competency in the dynamics of domestic abuse disclosure.* Sociology of Health and Illness, 2019. **41**(6): p. 1159-1174.
53. MacKichan, F., et al., *Why do patients seek primary medical care in emergency departments? An ethnographic exploration of access to general practice.* BMJ Open, 2017. **7**(4).
54. Maclean, A., et al., *Negotiation of collective and individual candidacy for long Covid healthcare in the early phases of the Covid-19 pandemic: Validated, diverted and rejected candidacy.* SSM - Qualitative Research in Health, 2023. **3**: p. 100207.
55. Mastrocola, E.L., A.K. Taylor, and C. Chew-Graham, *Access to healthcare for long-term conditions in women involved in street-based prostitution: a qualitative study.* Bmc Family Practice, 2015. **16**.
56. Mawson, R., *Understanding access to Sexual and Reproductive Healthcare (SRH) in General Practice using the Candidacy Framework.* 2022.
57. Methley, A.M., et al., *A qualitative study of patient and professional perspectives of healthcare services for multiple sclerosis: implications for service development and policy.* Health and Social Care in the Community, 2017. **25**(3): p. 848-857.
58. Methley, A., et al., *Meeting the mental health needs of people with multiple sclerosis: a qualitative study of patients and professionals.* Disability and Rehabilitation, 2017. **39**(11): p. 1097-1105.
59. Methley, A., *Health care services for multiple sclerosis: The experiences of people with multiple sclerosis and health care professionals.* 2015, University of Manchester.
60. Mughal, F., et al., *Experiences of general practice care for self-harm: A qualitative study of young people's perspectives.* British Journal of General Practice, 2021. **71**(711).
61. Novek, S. and V.H. Menec, *Age, Dementia, and Diagnostic Candidacy: Examining the Diagnosis of Young Onset Dementia Using the Candidacy Framework.* Qualitative Health Research, 2021. **31**(3): p. 498-511.
62. O'Brien, P., et al., *What are the patient factors that impact on decisions to progress to total knee replacement? A qualitative study involving patients with knee osteoarthritis.* BMJ Open, 2019. **9**(9).
63. Osborn, T.G., et al., *University students' access to mental health services: A qualitative study of the experiences of health service professionals through the lens of candidacy in England.* J Health Serv Res Policy, 2024. **29**(4): p. 230-239.
64. Peiris, D., et al., *Building better systems of care for Aboriginal and Torres Strait Islander people: Findings from the Kanyini health systems assessment.* BMC Health Services Research, 2012. **12**(1).

65. Pétrin, J., et al., *Healthcare access experiences of persons with MS explored through the Candidacy Framework.* Health and Social Care in the Community, 2021. **29**(3).
66. Rijken, M., et al., *Assessing the experience of person-centred coordinated care of people with chronic conditions in the Netherlands: validation of the Dutch P3CEQ.* Health Expectations, 2022. **25**(3): p. 1069-1080.
67. Sinnott, C., et al., *Understanding access to general practice through the lens of Candidacy: a critical review of the literature.* British Journal of General Practice, 2024. **26**(74): p. e683-e694.
68. Smyth, N., et al., *People from ethnic minorities seeking help for long COVID: a qualitative study.* British Journal of General Practice, 2024: p. 9.
69. Sundaresan, P., M.R. Stockler, and C.G. Milross, *What is access to radiation therapy? A conceptual framework and review of influencing factors.* Australian Health Review, 2016. **40**(1): p. 11-18.
70. Tarrant, C., et al., *'Falling through gaps': Primary care patients' accounts of breakdowns in experienced continuity of care.* Family Practice, 2015. **32**(1): p. 82-87.
71. Thomas, L., et al., *Health service brokerage to improve primary care access for populations experiencing vulnerability or disadvantage: A systematic review and realist synthesis.* BMC Health Services Research, 2019. **19**(1).
72. Tookey, S., et al., *Using the candidacy framework to understand how doctor-patient interactions influence perceived eligibility to seek help for cancer alarm symptoms: A qualitative interview study 11 Medical and Health Sciences 1117 Public Health and Health Services.* BMC Health Services Research, 2018. **18**(1).
73. Turk, F., et al., *Accessing care for Long Covid from the perspectives of patients and healthcare practitioners: A qualitative study.* Health Expectations, 2024. **27**(2).
74. Westlake, D., et al., *Terms of engagement for working with patients in a person-centred partnership: A secondary analysis of qualitative data.* Health & social care in the community, 2022. **30**(1): p. 330-340.
75. Whitford, K., et al., *Sexual and Reproductive Health Service Access among Aboriginal and Torres Strait Islander Young People in Regional NSW, Australia.* Health & Social Care in the Community, 2024. **2024**.
76. Williamson, L.E., K.E. Sleeman, and C.J. Evans, *Exploring access to community care and emergency department use among people with dementia: A qualitative interview study with people with dementia, and current and bereaved caregivers.* International Journal of Geriatric Psychiatry, 2023. **38**(7).
77. Wilson, N., et al., *UK healthcare services for people with fibromyalgia: results from two web-based national surveys (the PACFiND study).* BMC Health Services Research, 2022. **22**(1).
78. Worthing, K., et al., *Reluctance of general practice staff to register patients without documentation: a qualitative study in North East London.* British Journal of General Practice, 2022: p. BJGP.2022.0336.
